# Patients’ Experiences of a Sarcoma Diagnosis: A Process Mapping Exercise of Diagnostic Pathways

**DOI:** 10.3390/cancers15153946

**Published:** 2023-08-03

**Authors:** Sam Martin, Sigrún Eyrúnardóttir Clark, Craig Gerrand, Katie Gilchrist, Maria Lawal, Laura Maio, Ana Martins, Lesley Storey, Rachel M. Taylor, Mary Wells, Jeremy S. Whelan, Rachael Windsor, Julie Woodford, Cecilia Vindrola-Padros, Lorna A. Fern

**Affiliations:** 1 Rapid Research Evaluation and Appraisal Lab (RREAL), University College London, London W1W 7TY, UK; sam.martin@ucl.ac.uk (S.M.); sigrun.clark@ucl.ac.uk (S.E.C.); katie.gilchrist.19@ucl.ac.uk (K.G.); l.maio@ucl.ac.uk (L.M.); c.vindrola@ucl.ac.uk (C.V.-P.); 2 Sarcoma Unit, The Royal National Orthopaedic Hospital, Stanmore HA7 4LP, UK; craig.gerrand@nhs.net (C.G.); julie.woodford@nhs.net (J.W.); 3 Cancer Clinical Trials Unit, University College London Hospitals NHS Foundation Trust, London NW1 2PG, UK; 4 Department of Psychology, Anglia Ruskin University, Cambridge CB1 1PT, UK; lesley.storey@aru.ac.uk; 5 Centre for Nurse, Midwife and Allied Health Profession Research (CNMAR), University College London Hospitals NHS Foundation Trust, London NW1 2PG, UK; rtaylor13@nhs.net; 6 Nursing Directorate, Imperial College Healthcare NHS Foundation Trust, London W2 1NY, UK; mary.wells5@nhs.net; 7 Oncology Division, University College London Hospitals NHS Foundation Trust, London NW1 2PG, UK; 8 Paediatric Directorate, University College London Hospitals NHS Foundation Trust, London NW1 2PG, UK; rachael.windsor@nhs.net

**Keywords:** sarcoma, time to diagnosis, diagnostic timeliness, process mapping, teenagers and young adults, adolescence

## Abstract

**Simple Summary:**

Sarcomas are uncommon cancers growing in bones and soft tissue. As they are uncommon, we know less about them. Patients with sarcoma take longer to be diagnosed. This study examines what 78 people with different types of sarcomas told us about being diagnosed. We found out that approximately 7/10 people go to their General Practitioner (GP) first and do this within about 2 weeks of noticing something is wrong; however, those aged 13–24 years waited longer, at just over 4 weeks. Most people took approximately 3 months from noticing a symptom to being diagnosed, while those with soft tissue sarcoma took 5 months. More research is needed to help us diagnose sarcomas more quickly.

**Abstract:**

Patients with sarcoma often report prolonged time to diagnosis, which is attributed to the rarity of sarcoma and the low awareness of pre-diagnostic signs and symptoms. Aims: To describe patients’ experiences of pre-diagnostic signs/symptoms and pathways to diagnosis, including where help was sought, and the processes involved. Methods: Mixed methods involving quantitative, qualitative and inductive thematic analyses using novel process mapping of patient journey data, as reported by the patients. We examined the time from symptom onset to first professional presentation (patient interval, PI), first consultation to diagnostic biopsy, first consultation to diagnosis (diagnostic interval) and first presentation to diagnosis (total interval). Results: A total of 87 interviews were conducted over 5 months in 2017. Of these, 78 (40 males/38 females) were included. The sarcoma subtypes were bone (n = 21), soft tissue (n = 41), head and neck (n = 9) and gastro-intestinal (GIST; n = 7). Age at diagnosis was 13–24 (n = 7), 25–39 (n = 23), 40–64 (n = 34) and 65+ (n = 14) years. The median PI was 13 days (1–4971) and similar between sarcoma subtypes, with the exception of GIST (mPI = 2 days, (1–60). The longest mPI (31 days, range 4–762) was for those aged 13–24 years. The median diagnostic interval was 87.5 (range 0–5474 days). A total of 21 patients were misdiagnosed prior to diagnosis and symptoms were commonly attributed to lifestyle factors. Conclusions: Prolonged times to diagnosis were experienced by the majority of patients in our sample. Further research into the evolution of pre-diagnostic sarcoma symptoms is required to inform awareness interventions.

## 1. Introduction

Sarcomas are a rare and diverse group of cancers arising from bone and connective tissue. There are over 70 different subtypes that can all fall under the diagnosis of bone sarcoma or soft tissue sarcoma (STS) [1]. Sarcomas are heterogenous and differ greatly in the resulting morbidity and successful treatment rates [1]. For a significant proportion of patients, the physical burden of disease and treatment is high and, in many cases, accompanied by low expectations of long-term survival [2].

Patients with sarcoma frequently describe prolonged and complex pathways to diagnosis, but the impact on outcomes is not fully understood [3,4]. Times to diagnosis have been shown to adversely affect patient experiences in more common cancers [5]. The pathway from symptom onset to diagnosis is complex but recognised to be divided into distinct phases: the patient interval and the diagnostic interval [6,7,8]. The patient interval encompasses the time from the patient noticing the first symptom to the first consultation with a healthcare professional [6], whilst the diagnostic interval defines the time between the patient’s first encounter with a healthcare professional to receiving a cancer diagnosis [6].

The National Institute for Health and Care Excellence (NICE) recommends that adults with symptoms suggestive of sarcoma should be referred to specialists for further assessment within 2 weeks. For children and young people (those under 25 years), referral should be made within 48 hours [9,10]. However, the heterogeneity and ambiguity of symptoms that may be indicative of sarcoma often result in patients experiencing longer times to referral for specialist tests and diagnosis compared with more common cancers [4,11]. Prolonged times to diagnosis in rare cancers such as sarcoma can be attributed to the frequency with which healthcare professionals encounter a rare cancer diagnosis and less research detailing symptom profiles [4]. General Practitioners (GP) may only come across two sarcoma patients during their professional careers, and therefore may not be aware of symptoms potentially indicative of sarcoma [12].

There is room for improvement in the diagnostic experience of sarcoma patients. Sarcoma patients are more likely to report multiple GP visits and be dissatisfied with the time taken to see a hospital doctor compared with those with common cancers whose symptoms are more defined [4,11]. 

Early diagnosis of cancer is likely to improve patient experience and health-related quality of life (HRQoL), whilst prolonged time to diagnosis can lead to adverse clinical outcomes [13]. However, the relationship between time to diagnosis and outcome is complex and patients with an earlier diagnosis may experience higher mortality due to more aggressive disease [13,14]. HRQoL refers to an individual’s perception of the impact of their health status on their quality of life, including their physical, psychological and social functioning [2,15]. A study by Forster et al. reported that adolescents and young adults with longer times to diagnosis reported lower HRQoL and were more likely to be clinically anxious and depressed [16]. In patients with sarcoma, Soomers et al. examined the impact of prolonged time to diagnosis on HRQoL in the SURVSARC study, but no association was identified between the patient or diagnostic interval and HRQoL [2]. There was, however, an association between the perceived negative impact of diagnostic interval length on HRQoL and lower HRQoL scores [2]. In addition to clinical and quality of life outcomes, prolonged times to diagnosis have also been associated with legal action in sarcoma. The National Health Service (NHS) litigation costs for extremity sarcoma between 1995 and 2010 were £4.4 million, and 89% of these cases were related to time to diagnosis [17]. 

Process mapping of patient journey data (from electronic and hospital databases) has been used in previous healthcare research with the aim of better understanding the experience patients in various health settings [18,19,20]. Rojas et al. [18] have referred to process mining as a way of extracting data related to an event, and they describe models as orders in which activities have occurred within healthcare. Much has been written about the different types of process mapping software that can be utilised to map patient journeys [21,22,23,24].

We aimed to use a large qualitative dataset to describe pre-diagnostic signs/symptoms and pathways to diagnosis, including where help was sought and what processes were involved prior to the diagnosis. Our intention was to visualise the journey of experiences encountered by patients up to their diagnosis and to identify any commonalities between subgroups of patients with sarcoma.

## 2. Materials and Methods

We adopted a mixed methods approach involving quantitative, qualitative and inductive thematic secondary analyses of 87 interviews conducted over five months in 2017 [25]. In this current study, we have opted to use the interactive process mapping software Celonis Process Mapping 4.7.2 [23,24]. The Celonis platform utilises process analytics algorithms in a way that allows us to map and analyse patients’ health pathways/journeys. Our process mapping analysis was informed by the categorisation of patients’ accounts of appointment frequency, timeline and type, as well as definitive sarcoma diagnosis. 

### 2.1. Data Collection 

The primary interview data were collected by two experienced researchers (AM and RT). The purpose of the interviews was to inform an outcome measure for sarcoma [26]. Participants were recruited from 12 hospitals across England and Scotland and also through social media. Newsletters from Sarcoma UK and Bone Cancer Research Trust facilitated the participation of patients from across the United Kingdom. Eligibility criteria were broad and included any sarcoma and time from diagnosis, aged ≥ 13 years and able to communicate verbally or in writing in English. Sampling considered socio-demographic factors such as age and gender, location of care, sarcoma type, primary site, treatment type (surgery, radiotherapy, chemotherapy), time since diagnosis, and other factors (e.g., recurrence, clinical trial participation, metastases). Healthcare professionals at recruiting sites issued patient information leaflets and obtained written consent from patients. For those < 16 years, additional consent was obtained from parents/legal guardians. Consents were emailed to the study office. Patients who contacted the study office directly via social media/newsletter content were given verbal information about the study, and if interested, were sent information sheets and a consent form. Telephone interviews were arranged once completed consent forms had been received by the study team. Consent interviews were audio recorded. Interviews were between 26 and 96 min.

A secondary analysis of the transcripts was conducted due to the extent of detail patients shared about their diagnostic experiences. An initial round of secondary data analysis was conducted by the research team (AM, LF and RT). Additional secondary data analysis was conducted by five researchers (SM, SEC, KG, LM, CV) from the Rapid Research, Evaluation and Appraisal Lab (RREAL) at University College London, between December 2021 and June 2022. 

### 2.2. Ethical Approvals

The study was approved by the Health Research Authority (HRA) (London Stanmore REC ref: 16/LO/2152). Patients consented to the interview before it started and were aware they could stop and leave the interview at any time. No such instances occurred. 

The interview data were grouped into four age cohorts: 13–24 years, 25–39 years, 40–64 years and 65+. These groupings were chosen based on the classification of teenagers and young adults in England (13–24 years), European and United States definitions of young adults (25–29) and then older adults and the elderly (>65 years). 

### 2.3. Secondary Data Analysis 

The secondary data analysis was conducted in Microsoft Excel using pre-defined categories such as appointment frequency, timeline and type, as well as definitive sarcoma diagnosis. Inductive thematic analysis was conducted by three researchers (KG, SEC, LM) to identify statements within transcripts related to each pre-defined category. Two team members (LF and RW), who included a clinician, were consulted for verification based on the information within the transcript. This was conducted for wider consultation and consensus on whether participants had true medical misdiagnosis, or if the experiences described were logical diagnostic procedures carried out in the processes/steps of elimination needed to make a final diagnosis. For some interviews, it was not possible for us to determine if there had been a misdiagnosis. 

### 2.4. Process Mapping: Data and Appointment Log Construction

Data from the qualitative analysis were extracted from the spreadsheet in CSV (comma-separated value) format and reformatted to encompass information about clinical and appointment episodes for each patient. To create the appointment activity log, we considered the following features from the qualitative data:

1. Patient ID: corresponds to the patient case number from Celonis.
2. Activity: refers to the type of appointment involved in patients’ interactions with clinical services.
3. Timestamp: refers to the date the first symptom was noticed by the patient.
4. Age cohort: age at time of diagnosis.
5. Gender: patients’ declared gender.
6. Symptom: nature of initial symptom(s).
7. Misdiagnosis: if the patient experienced a medical misdiagnosis.
8. Final Diagnosis: the final sarcoma diagnosis received by the patient.
9. Diagnosis type: soft tissue, bone, head and neck (H and N) and Gastrointestinal Stromal Tumours (GIST). Clinically, H and N and GIST behave distinctly, hence we have classified them separately for this analysis.
10. NHS or Private: the type of public/private health service used for each appointment.
11. Recurrence: if the sarcoma diagnosis was a recurrence or a primary diagnosis.

**Figure 1:**
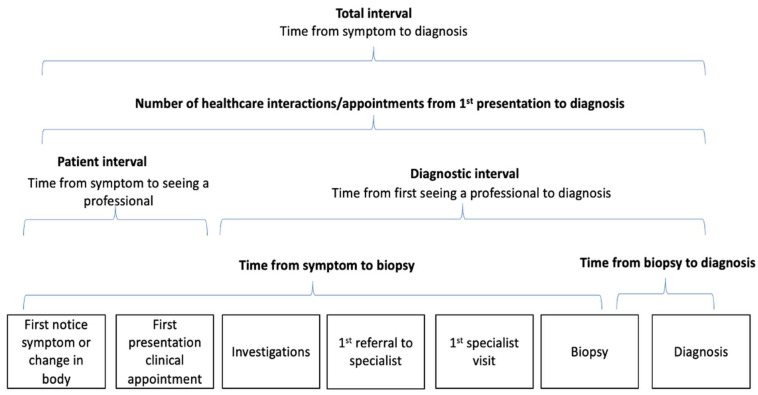
Illustrates how the interview data were categorised into subintervals based on previously described models.

A process explorer algorithm within Celonis was used to obtain an analysis model that shows the different touchpoints of the patient’s journey. A process explorer algorithm uses an exploratory approach by first analysing the most frequent activities and mapping any related connections [27]. The Celonis process explorer was used to calculate the median patient and diagnostic interval as well as subintervals such as the interval lengths between hospital biopsy and final diagnosis [23]. Intervals and subintervals were based on previous research and pathways described by Ossen et al. and are shown in Figure 1 and Table 1 [7,8]. Patients were categorised based on sarcoma type and if they were initially misdiagnosed. Sarcoma subtypes were bone, soft tissue sarcoma, head and neck and GIST. Head and neck sarcomas were grouped into a separate category as they tend to present through different pathways. Appointments were categorised by the type of healthcare facility they were in—either within the UK NHS or, where an appointment was in the UK but under private medical care, these were tagged as “(Private)” and where appointments were attended outside the UK, the name of the country was also tagged to the appointment touchpoint (e.g., Spain).

### 2.5. Assumptions and Logic 

A number of logical decisions were applied to the data, for example, geographical location, appointment type, number of appointments, treatments and number of recurrences. A number of assumptions were also used. These included the number of appointments, interactions with GPs, location of appointments and structure of diagnosis-sharing. A detailed list of logic and assumptions applied can be found in Table 1. 

The time from symptom onset to diagnosis is defined in Celonis as the total throughput time between certain activities/appointments. Within the period from symptom onset to diagnosis, the time between symptom start and appointment 1 is defined as the patient interval, and the time between appointment 1 and diagnosis is defined as the diagnostic interval. We report median and range throughout the manuscript and present descriptive statistics appropriate for quantitative data extracted from the interviews.

### 2.6. Missing Data

Nine participants were not included in the process mapping analysis due to missing data on patient interval and decision to see a GP, or on the diagnostic interval in terms of the number of appointments with HCPs before diagnosis. Therefore, 78 participants were included in the process mapping analysis. 

### 2.7. Clinical Confirmation of Sarcoma Subtype

A total of 16 participants did not state the sarcoma subtype they had been diagnosed with. The clinicians were able to deduce the sarcoma subtype based on details in the transcripts about procedures and descriptions of their conditions and treatments. Of the 16 cases, the clinicians were able to identify 3 bone sarcomas and 11 soft tissue sarcomas but were unable to confirm the diagnosis of the remaining 2 due to insufficient detail on sarcoma type in the interview transcript. 

### 2.8. Identifying Those with Misdiagnosis

The clinicians reviewed 26 cases to determine whether the interviewees’ description of their pathway to diagnosis suggested misdiagnosis or whether it appeared to be a reasonable pathway of diagnostic elimination. Of these, 21 were confirmed as misdiagnoses, 4 were considered reasonable pathways of diagnostic decision-making and 1 could not be confirmed as misdiagnosed, although it was still included based on sarcoma diagnosis. Details of the clinician’s decision-making and the types of diagnoses can be found in File S1.

### 2.9. Thematic Analysis 

Thematic analysis was conducted on the transcripts from bone sarcoma patients who experienced the longest time from symptom onset to diagnosis and misdiagnoses to identify key themes and patterns in the data that may contribute to longer times to diagnosis [25]. 

## 3. Results

### 3.1. Demographics

A total of 40 males and 38 females were included in the analysis. Of these, 21 were diagnosed with bone sarcoma, 41 with soft tissue sarcoma, 9 with head and neck sarcoma, and 7 with GIST. Age distribution was 13–24 years (n = 7), 25–39 years (n = 23), 40–64 years (n = 34) and 65+ years (n = 14). Table 2 shows the patients’ demographics. 

### 3.2. Patient Intervals

Overall, the median patient interval (mPI) was 13 days (range 1–4971) and was similar between males and females and across sarcoma subtypes, with the exception of GIST (mPI = 2 days (range 1–60)). By age, the longest mPI was for those aged 13–24 years (31 days, range 4–762) and notably for those aged 13–24 with soft tissue sarcoma mPI (61, range 14–762), although numbers are small within this subgroup (n = 3). Table 3 shows mPI and diagnostic intervals for all subgroups. 

### 3.3. First Presentation to Biopsy and Diagnosis 

**Figure 2:**
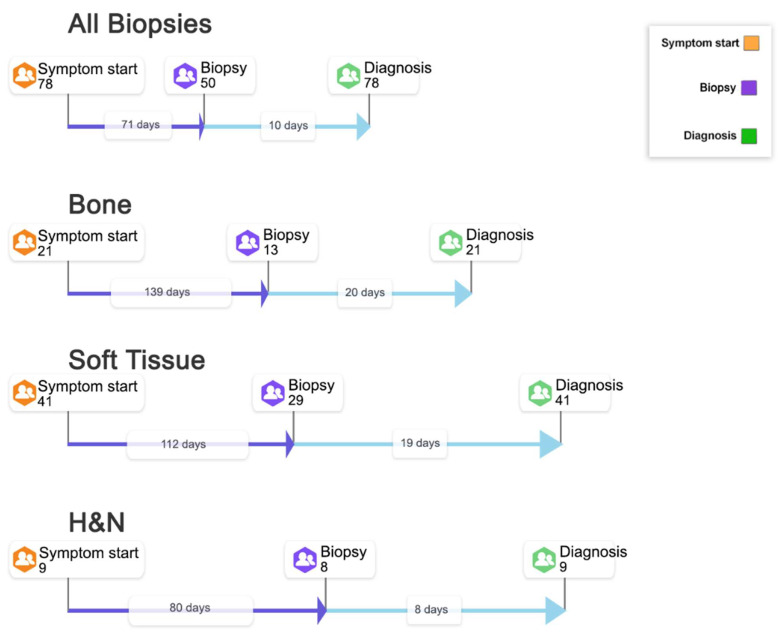
Symptom onset to biopsy: cases from symptom onset through to biopsy and onto final diagnosis (Median in days). Legend: Information from 78 interview participants has been collated to develop a pathway to show the number of cases from symptom start through to biopsy and onto final diagnosis. Of the 78 participants, 50 described having a biopsy before the final diagnosis. STS: soft tissue sarcoma; H&N: head and neck.

A total of 50 patients (64%) reported receiving a biopsy prior to the final diagnosis. While some patients did not specifically mention a tissue biopsy, it is presumed they all had a biopsy. It should be noted that only one GIST patient reported a biopsy; however, this patient had >5000 days interval, which potentially skews the overall biopsy section. For this reason, we have removed GIST from the overall median and have not included this in the biopsy section. Therefore, the median time from symptom onset to biopsy was 71 days (10.1 weeks). Following biopsy, the median time to final diagnosis was 10 days (1.4 weeks); this was the shortest interval in the pathway across all sarcoma types (Figure 2 shows the median period pathways for all biopsies and individual sarcoma types). 

### 3.4. Diagnostic Interval 

**Figure 3:**
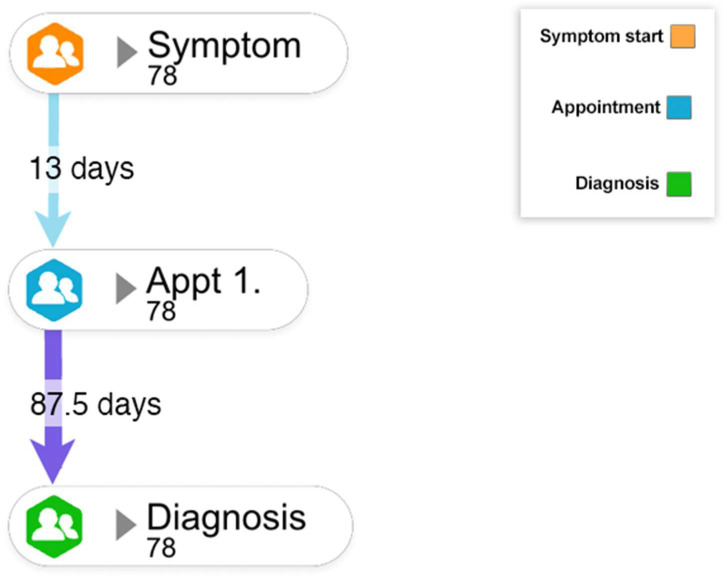
Median time (in days) from symptom onset to diagnosis. Legend: Information from 78 interview participants was collated to develop a pathway that captures the median number of days between the first healthcare appointment and diagnosis. Based on this information, the median patient interval and the diagnostic interval can be calculated.

The median diagnostic interval for all 78 patients was 87.5 days (range 0–5474), generally longer than the patient interval across all groups (See Table 3 and Figure 3). This varied by sarcoma type and was shortest for H and N sarcoma (57 days, range 8–2314) and longest for bone sarcoma (127 days, range 2–799). The median diagnostic interval was 109.5 days (1–2314) for females and 73 days (0–5474) for males. 

### 3.5. Total Intervals (Symptom Onset to Diagnosis)

The median time from symptom onset to diagnosis varied by sarcoma type and was longest for soft tissue sarcoma at 145 days (4–5693) and shortest for GIST at 25 days (1–5479) (See Table 4). 

### 3.6. Symptoms and Diagnostic Pathway Characteristics

The most frequently reported symptoms varied by sarcoma type; however, pain and/or a lump were most commonly reported across the four subtypes (Table 4).

#### 3.6.1. Bone Diagnosis Pathway Characteristics 

Bone sarcomas were diagnosed across all age cohorts. A higher proportion of males were diagnosed with bone sarcoma compared with females (males: 14, females: 7), and the majority of those with bone sarcoma were aged 25–39 at diagnosis (n = 10/21). The most common symptom experienced by patients was pain, most prevalent in the lower parts of the body. Prior to being diagnosed with bone sarcoma, seven of the patients, the majority of whom were male (males: 5, females: 2), received a misdiagnosis (refer to File S1). There were three cases of recurrence. Further details can be found in Table 2, Table 3 and Table 4.

#### 3.6.2. Soft Tissue Diagnosis Pathway Characteristics 

Soft tissue sarcoma was the most common sarcoma diagnosed across all ages. The majority were 40–64 years at diagnosis and mainly female (males: 18, females: 23). The main symptom experienced was a lump. A total of nine patients were initially misdiagnosed. We were unable to confirm a misdiagnosis for one patient. Misdiagnosis in this group was more common in females (males: 2 females: 7) (refer to File S1). Soft tissue sarcomas had the longest median time from symptom onset to diagnosis (145 days, range 4–5693). There were nine cases of recurrence. Further details can be found in Table 2, Table 3 and Table 4.

#### 3.6.3. Head and Neck Diagnosis Pathway Characteristics 

A smaller proportion of the patients were diagnosed with head and neck sarcoma, some of whom were identified as bone or soft tissue subtypes. Most of the diagnoses were in females (males: 3, females: 6) and across all four age cohorts, with the majority in the 40–64 age cohort (n = 5). The symptoms experienced by patients in this group were largely varied, including issues affecting the eyes, mouth and jaw. There was one case of recurrence, and three patients experienced misdiagnosis (males: 1, females: 2) (refer to File S1). 

#### 3.6.4. Gastrointestinal Tumour Diagnosis Pathway Characteristics 

GIST was the least common form of sarcoma diagnosed, as would be expected based on incidence. Most of the diagnoses were in males (males: 5, females: 2). Patients in this group were mainly in the 40–64 age cohort, with two older patients in the 65+ age cohort. The most common symptoms experienced by patients with this diagnosis were pains, lumps, reflux in the stomach, swelling and blood in the stool. There were two patients who experienced recurrence, and two of the patients (male) were misdiagnosed (given treatment for reflux/diagnosed as having a hernia) prior to their diagnosis (refer to File S1). Further details can be found in Table 2, Table 3 and Table 4. 

### 3.7. Misdiagnosis 

**Figure 4:**
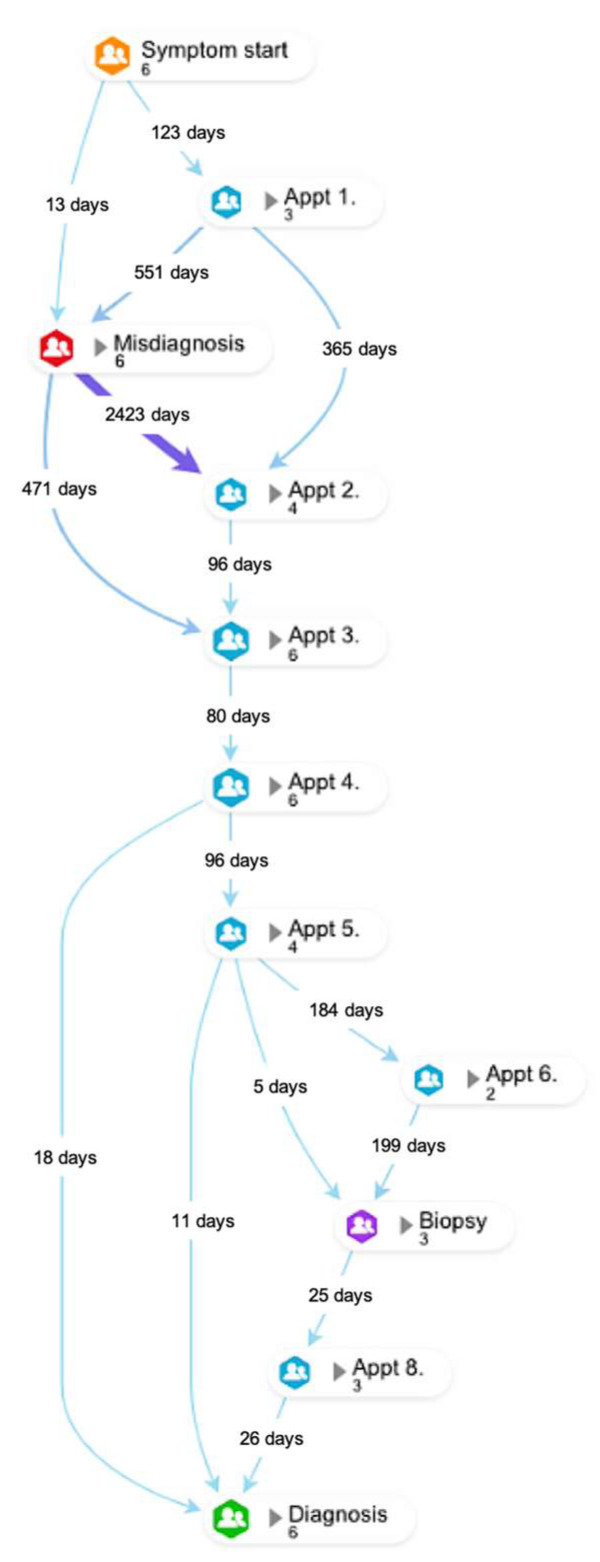
Diagnostic pathway of the six patients experiencing the longest times from symptom onset to diagnosis.

There were 21 patients who were misdiagnosed—they were initially diagnosed or treated for other conditions before being diagnosed with sarcoma. Further details of these cases can be found in File S1. The diagnostic pathways of the six patients who received a misdiagnosis and had the longest throughput times can be found in Figure 4.

Key quotations from some of the bone sarcoma patients who had received misdiagnoses and the longest throughput times are found below. The quotes illustrate the symptoms being attributed to lifestyle factors such as sports, gym or work, alongside more commonly seen conditions such as infection.

“Initially, it was believed that I had cartilage damage in my knee, in the joint, because several years ago previous I’d been playing squash and experienced a lot of pain in that knee when I’d gone for a shot and lunged.” … “Was referred to physio, who just gave me, sort of, exercises to strengthen joints.”

(Male, 25–39)

“I kept going to the doctors because I just didn’t feel any better, you know. When I first told them about the lump, all he did was tell me to go to the physio and just said it was just an infection.”

(Male, 65+)

“I kept going to the doctors and he just kept giving me painkillers which weren’t really resolving the issue.” … “Had a [private] consultation to investigate the knee with an MRI on the knee and there was nothing wrong with it. My consultant said, ‘Oh well, just carry on. There’s nothing wrong with the knee.’” … “I think my GP let me down a bit…with the initial diagnosis and not listening properly and having to fight with him to get referrals. Literal verbal fighting to get referrals. That was probably the hardest bit.”

(Male, 40–64)

“I went to see the GP in 1982, I can’t remember on how many occasions, but it might’ve been on two or three occasions, to try say something was wrong… He said, ‘It’s probably just strained it playing squash,’ and prescribed some painkillers, and suggested I don’t play squash for two weeks.”

(Male, 65+)

“My GP, since 2013 until 2015, was explaining that the pain might be-, the main reason of the pain is tendinitis or worked too hard in the gym… So, I should just take some painkillers and some creams, anti-inflammatory creams, to relieve the pain, and that was it.” … “I felt definite, huge anger, because the main reason why this happened is because of the GP, who couldn’t treat me two years ago in a proper way. He didn’t listen to my concerns about my shoulder situation.”

(Male, 25–39)

## 4. Discussion

We carried out secondary analysis of qualitative interview data using a novel methodology to map pathways from symptom onset to diagnosis for patients with sarcoma. 

The characteristics of the diagnostic pathway differed between sarcoma types, similar to other reports [3,6,28,29]. Patients, particularly men, with bone sarcoma had the longest times to diagnosis and were the most likely to have been misdiagnosed (refer to File S1). Misdiagnosis occurred due to symptoms being attributed to active lifestyles, for example, participating in activities such as squash and kickboxing. Participants mentioned not being taken seriously and expressed frustrations with the length of the diagnostic interval. Previous studies reported similar findings, where individuals with bone or soft tissue tumours were misdiagnosed with sports injuries, most commonly around knee joints [30,31]. In a recent study of 1117 respondents (of all ages) conducted by Sarcoma UK, 22% were told at their first appointment that their symptoms were not serious, and 17% were given treatment for another condition after the first appointment [12].

We found no differences in patient interval based on gender, similar to other reports [6,28]. However, comparison with other subgroups is difficult due to differences in the methodologies and time intervals used. The study by Drabbe et al. [6] reported long diagnostic intervals (≥1 month) in 55% of the patients and very long intervals (≥3 months) in 28% of the patients. Our patient intervals were shorter in comparison, with the exception of soft tissue sarcoma.

Soft tissue sarcoma was the most common type of diagnosis and had the longest time from symptom onset to diagnosis. Previous studies have highlighted the difficulty in identifying sarcomas, as only 1 in 100 soft tissue lumps are malignant. This means that GPs may often believe a lump is benign, contributing to the longer time from symptom onset to diagnosis [29]. This is confounded by the low positive predictive value of potential sarcoma alert symptoms. The study by Dyrop et al. showed that only 18% of patients referred on a fast-track suspected sarcoma pathway had a final diagnosis of sarcoma [28]. Recurrence was more common in patients with soft tissue sarcoma. Previous studies have shown that approximately 11–14% of treated patients experience local recurrence [32]. For most of the patients in our sample, there was often a quick route to diagnosis once biopsy was completed. This has been found in previous research, highlighting the importance of ordering imaging and biopsies for persisting or growing soft tissue lumps [33].

There were relatively few specified diagnoses of head and neck sarcomas. For this sarcoma, the routes to diagnoses were relatively short, with a median of 83 days, compared with the other sarcoma types. Typically, these patients present through different routes. A previous cancer diagnosis in two participants may have expedited diagnosis, as both medical professionals and patients were more vigilant. 

In our study, referral to a specialist for symptoms, which might be a sarcoma, exceeded the NICE recommendation of 2 weeks for most patients. Our findings on misdiagnosis characteristics and routes to diagnosis are in keeping with other studies showing sarcoma patients experience multiple healthcare visits, are treated for other conditions and face diagnostic intervals >1 month [4,6,11,12]. 

The difficulty in diagnosing rare cancers, particularly sarcoma, is reported and discussed above. Our pathway mapping supports interventions to raise patient and professional awareness and highlights the need for vigilance and intervention around non-resolving sports injuries and imaging and biopsy of growing soft-tissue lumps. Unlike most cancers where healthcare interactions start with the GP, patients may present to other health professionals first. Professionally, awareness amongst physiotherapists as well as GPs is relevant for sarcomas. Particular patient groups are identified for targeted interventions, for example, longer patient intervals are evident in teenagers and young adults and longer diagnostic intervals are evident in females. 

### 4.1. Future Research 

This study adds value to existing knowledge on pathways to sarcoma diagnosis; however, further studies designed specifically to quantify and describe the diagnostic pathway in sarcoma are needed. To increase the robustness of data, future studies should be designed to include electronic health records and GP records alongside interviews. Understanding the impact of diagnostic pathways on HRQoL and other outcomes would also be of value. 

### 4.2. Strengths and Limitations 

Compared with existing process mapping literature in oncology, we included multiple cases (n = 78) compared with an average of 9 [18]. However, our study was based on participants’ accounts of their experiences of healthcare, whereas other studies have used electronic health records. The broad inclusion criteria for participants meant our data reflected a range of disease types and age experiences. The 78 interviews offered considerable detail on diagnostic experiences. 

This study has some limitations. In addition to recruitment through sarcoma clinics, patients could self-select for inclusion via social media and invites through newsletters from Sarcoma charities. This population may not be as representative of the general sarcoma population, as they are more motivated to take part in research. A number of participants were two years beyond their diagnosis; this increased the risk of recall bias and possible distortion of the sequence and timing of events. Furthermore, the original interviews were not conducted primarily for process mapping or examination of routes and times to diagnosis, therefore transcripts offered different levels of detail. Supplementation of the patient experiences with medical records would have allowed greater insight and added depth to the diagnostic processes; however, we did not consent to this. In some manuscripts, important information on dates and locations of appointments and diagnoses was missing, and therefore could not be included in the analysis. Based on the logic that was applied to scenarios within Table 1, there were instances where the exact number of appointments was not defined and instead referred to as ‘many’ or ‘multiple’ appointments. Where we could not define the exact number, we categorised them in the process mapping as equal to or greater than two appointments. 

## 5. Conclusions

This is the first study to use novel process mining pathways to examine diagnostic experiences in patients diagnosed with sarcoma. Most patients faced diagnostic intervals that exceeded the current diagnostic targets outlined in the policy. We and others have previously shown a possible link between longer diagnostic intervals and diminished patient experience, and therefore further work on the evolution of sarcoma symptoms to support physicians in prompt identification and referral of patients with suspected sarcoma is a priority. 

## Figures and Tables

**Table 1:** Logic and assumptions applied to transcripts and the subinterval definitions.

Logic applied (considered as a general ‘rule of thumb’)	1. Appointments included all countries (noted where not UK).
	2. Appointments included in-person, telephone and letters (where the diagnosis was written).
	3. Where participants mentioned ‘multiple/many/several’ ‘GP apps/ultrasounds/MRIs’ etc., these were counted as ≥2 (equal or greater than 2).
	4. MRIs/CTs/biopsies performed following the confirmed diagnosis were not included in appointment counts but were included in the treatment phase.
	5. Total appointment count = number of appointments + 1 (diagnosis), with the exception that there were some with the tag ≥ 2
	6. Where participants described ‘multiple/many/several’ recurrences with no reference to dates or number of appointments, these were counted as one recurrence.
	7. Filters for ‘who/when/where/how’. We have listed the specific scan (MRI/CT/Ultrasound) where specified. If the participant simply said ‘scan’, we listed ‘scan’.
	8. Data for six participants were not included in process mapping due to missing data.
Assumptions applied (how we have treated data in the absence of details)	1. Where several procedures were mentioned (MRI, X-ray, Biopsy) but not stated if they were different times/days, assumed they were different appointments unless stated otherwise and therefore treated as different ‘touch points/occasions’ and counted as separate appointments.
	2. When patients referred to ‘they’ for appointments before scans or biopsy procedures, it was assumed to be in relation to GPs.
	3. Assumed MRIs/CTs/Biopsies were in hospitals unless otherwise stated.
	4. Assumed the diagnosis/sharing of results was a separate appointment unless stated otherwise.
	5. In one transcript we have assumed the patient is male, based on a mistake in the transcript (assumed the male moderator should have read male respondent).
Subinterval definitions	1. Time from symptom onset to diagnosis: the total time of all intervals.
	2. Patient interval: the time between symptom start and the first appointment with healthcare professionals.
	3. Diagnostic interval: the time between appointment 1 and diagnosis. This interval included primary and secondary care, whereby it has been assumed that procedures such as biopsies and scans would be classified as secondary care.
	4. Time from presentation to biopsy: the time between 1st appointment and the first biopsy.
	5. Time from biopsy to diagnosis: time between biopsy and sarcoma diagnosis.

GP: General Practitioner; MRI: Magnetic Resonance Imaging; CT: computerized tomography.

**Table 2:** Patient demographics.

		BS (n = 21)	STS (n = 41)	H and N (n = 9)	GIST (n = 7)
Gender	Male	14 (67.7)	18 (43.9)	3 (33.3)	5 (71.4)
n (%)	Female	7 (33.3)	23 (56.1)	6 (66.7)	2 (28.6)
Age in years	13–24	3 (14.3)	3 (7.3)	1 (11.1)	0 (0.0)
n (%)	25–39	10 (47.6)	11 (26.8)	2 (22.2)	0 (0.0)
	40–64	5 (23.8)	19 (46.3)	5 (55.6)	5 (71.4)
	65+	3 (14.3)	8 (19.5)	1 (11.1)	2 (28.6)

BS: Bone Sarcoma; STS: soft tissue sarcoma; H and N: head and neck; GIST: gastrointestinal stromal tumour.

**Table 3:** Patient Interval and diagnostic interval by patient groups.

		Patient Interval (Days)	Diagnostic Interval (Days)
		Median, Range	Median, Range
Sarcoma	All	13 (1–4971)	87.5 (0–5474)
	Bone (n = 21)	13 (1–372)	127 (2–799)
	STS (n = 41)	14 (1–4971)	86 (1–1276)
	H and N (n = 9)	14 (1–365)	57 (8–2314)
	GIST (n = 7)	2 (1–60)	13 (0–5474)
Male (n = 40)	All	13 (1–4971)	73 (0–5474)
	Bone (n = 14)	13 (1–372)	131 (10–799)
	STS (n = 18)	23.5 (1–4971)	48 (3–900)
	H and N (n = 3)	26 (2–38)	30 (8–88)
	GIST (n = 5)	2 (1–5)	22 (0–5474)
Female (n = 38)	All	13.0 (1–730)	109.5 (1–2314)
	Bone (n = 7)	14 (4–36)	112 (2–223)
	STS (n = 23)	13 (1–730)	120 (1–1276)
	H and N (n = 6)	10.5 (1–365)	87.5 (15–2314)
	GIST (n = 2)	30.5 (1–60)	7 (1–13)
13–24 years (n = 7)	All	31 (4–762)	57 (2–639)
	Bone (n = 3)	31 (4–36)	14 (2–128)
	STS (n = 3)	61 (14–762)	229 (21–639)
	H and N (n = 1)	4 (4–4)	57 (57–57)
	GIST (n = 0)	n/a	n/a
25–39 years (n = 23)	All	13 (1–372)	120 (10–1276)
	Bone (n = 10)	13 (1–372)	123.5 (10–799)
	STS (n = 11)	13 (1–366)	120 (26–1276)
	H and N (n = 2)	20 (14–26)	108 (88–128)
	GIST	n/a	n/a
40–64 years (n = 34)	All	10 (1–1827)	27 (0–5474)
	Bone (n = 5)	13 (1–365)	95 (61–239)
	STS (n = 19)	13 (1–1827)	26 (1–900)
	H and N (n = 5)	38 (1–365)	22 (8–2314)
	GIST (n = 5)	2 (1–5)	2 (0–5474)
65+ years (n = 14)	All	11 (1–4971)	148.5 (3–1135)
	Bone (n = 3)	11 (6–32)	432 (146–662)
	STS (n = 8)	14 (1–4971)	118.5 (3–1135)
	H and N (n = 1)	7 (7–7)	118 (118–118)
	GIST (n = 2)	31 (2–60)	568 (13–1123)

STS: Soft tissue sarcoma; H and N: Head and Neck; GIST: gastrointestinal stromal tumour.

**Table 4:** Commonly reported symptoms and total intervals by sarcoma type (time from symptom onset to diagnosis).

		Bone (n = 21)	STS (n = 41)	H and N (n = 9)	GIST (n = 7)
Main symptom(s)	Pain	14 (66.7)	7 (17.1)	2 (22.2)	2 (28.6)
n (%)	Lump/swelling	3 (14.3)	25 (61.0)	4 (44.4)	2 (28.6)
	Other clinical examination ^1^	2 (9.5)	0 (0.0)	0 (0.0)	0 (0.0)
	Other ^2^	2 (9.5)	8 (19.5)	3 (33.3)	3 (42.9)
	Unknown	0 (0.0)	1 (2.4)	0 (0.0)	0 (0.0)
Recurrence	Yes	3 (14.3)	9 (22.0)	1 (11.1)	2 (28.6)
n (%)	No	18 (85.7)	32 (78.0)	8 (88.9)	5 (71.4)
Misdiagnosis	Yes	7 (33.3)	9 (22.0)	3 (33.3)	2 (28.6)
n (%)	No	14 (66.7)	31 (75.6)	6 (66.7)	5 (71.4)
	Unsure	0 (0.0)	1 (2.4)	0 (0.0)	0 (0.0)
Time from symptom onset to diagnosis	Median days (min–max)	136 (23–803)	145 (4–5693)	83 (16–2679)	25 (1–5479)

STS: Soft tissue sarcoma; H and N: Head and Neck; GIST: gastrointestinal stromal tumour. ^1^ found as part of clinical examination for a different problem, for example, a fracture. ^2^ examples include continuous cough, general malaise, ovarian cyst and gastrointestinal reflux.

## Data Availability

Data are available on request from the authors.

## Supplementary Materials

The following supporting information can be downloaded at: https://www.mdpi.com/article/10.3390/cancers15153946/s1, File S1: Details of patients who were confirmed as being misdiagnosed.
