# Supplementary material for: Patients’ Experiences of a Sarcoma Diagnosis: A Process Mapping Exercise of Diagnostic Pathways
Source: Cancers (Basel). 2023 Aug 3;15(15):3946. doi: 10.3390/cancers15153946 (PMC10417695; doi:10.3390/cancers15153946)
Supplement: Supplementary file 1 [file cancers-15-03946-s001.zip › cancers-2505170-supplementary.pdf]

Supplementary Material

File S1 – Details of patients who were confirmed as being misdiagnosed

ID	Gender	Age group	Symptom (body location)	Recurrence	Details	Time from symptom onset to diagnosis in days
Bone	Female	25-39	Pain (thigh)	No	GP refused MRI, (patient went private)	96
Bone	Female	40-64	Pain (knee)	No	Assumed a broken bone	109
Bone	Male	25-39	Pain (knee)	No	Referred/provided physiotherapy	451
Bone	Male	65+	Lump (hip)	No	Referred/provided physiotherapy	464
Bone	Male	40-64	Pain (knee)	No	Prescribed pain killers	492
Bone	Male	65+	Pain (knee)	No	Assumed loose ligaments & fracture	673
Bone	Male	25-39	Pain (shoulder)	No	Assumed tendonitis	803
Soft tissue	Female	25-39	Bruise & body changes	No	Assumed a benign tumour	133
Soft tissue	Female	13-24	Pain (gynaecological and leg)	No	Received negative tumour markers	243
Soft tissue	Male	65+	Pain (back)	No	Assumed was lipoma	244
Soft tissue	Female	40-64	Lump (shin)	No	Did not advise MRI	267
Soft tissue	Female	40-64	Ill health	Yes	Assumed fibroids and advised hysterectomy	329
Soft tissue	Female	25-39	Pain (lower back)	Yes	Assumed growing pains	768
Soft tissue	Male	40-64	Pain (shoulder)	No	Assumed scar tissue	904
Soft tissue	Female	65+	Lump (chest)	No	Assumed fatty lump and advised ibuprofen	1,166

Soft tissue	Female	25-39	Lump (cyst)	No	Assumed cyst	1,277
Soft tissue	Female	25-39	Not mentioned	Yes	Unsure if rectus sheath hematoma was a misdiagnosis	505
Head and neck	Male	40-64	Tingling (lip)	Yes	Assumed chondrosarcoma	32
Head and neck	Female	25-39	Lump (gum)	No	Pathology not done on lump	142
Head and neck	Female	40-64	Lump/tumour (jawbone)	No	Initially thought non-malignant	2,679
GIST	Male	40-64	Large swelling	No	Large swelling assumed a hernia	4
GIST	Male	40-64	Reflux/stomach issues	No	Prescribed treatment for reflux	5,479

GP: General Practitioner; MRI: Magnetic Resonance Imagining; GIST: Gastro-intestinal imaging
